# Loops and Building Blocks: a Knowledge co-Production Framework for Equitable Urban Health

**DOI:** 10.1007/s11524-021-00531-4

**Published:** 2021-03-18

**Authors:** Camilla Audia, Frans Berkhout, George Owusu, Zahidul Quayyum, Samuel Agyei-Mensah

**Affiliations:** 1 Department of Geography, School of Global Affairs, Faculty of Social Science and Public Policy, King’s College London, Strand, London, WC2R 2LS UK; 2 Institute of Statistical, Social and Economic Research (ISSER), University of Ghana, Accra, Legon Ghana; 3 James P Grant School of Public Health, BRAC University, Dhaka, Bangladesh; 4 Department of Geography and Resource Development, University of Ghana, Accra, Legon Ghana

**Keywords:** Urban health, Co-production of knowledge, Policy, Impact, Equity

## Abstract

This paper sets out a structured process for the co-production of knowledge between researchers and societal partners and illustrates its application in an urban health equity project in Accra, Ghana. The main insight of this approach is that research and knowledge co-production is always *partial*, both in the sense of being incomplete, as well as being circumscribed by the interests of participating researchers and societal partners. A second insight is that project-bound societal engagement takes place in a broader context of public and policy debate. The approach to co-production described here is formed of three recursive processes: co-designing, co-analysing, and co-creating knowledge. These ‘co-production loops’ are themselves iterative, each representing a stage of knowledge production. Each loop is operationalized through a series of research and engagement practices, which we call *building blocks*. Building blocks are activities and interaction-based methods aimed at bringing together a range of participants involved in joint knowledge production. In practice, recursive iterations within loops may be limited due of constraints on time, resources, or attention. We suggest that co-production loops and building blocks are deployed *flexibly*.

## Introduction

Health equity research highlights how economic, social, and health policy choices may result in uneven health outcomes across populations [1]. This is particularly visible in cities, where differential exposure to health risks is compounded by pre-existing and interconnected vulnerabilities of urban populations. Despite increasing understanding about health in cities and its relationship with risks, such as social exclusion, poverty, housing, sanitation, and environmental quality, there continues to be a gap between available and actionable knowledge, resulting in decision-makers not acting on best evidence, even where policy objectives are agreed. How can research and policy evolve more closely, resulting in options that are useful, usable, and used by decision-makers? This paper presents a methodological framework, drawing on social science literatures, for co-production of knowledge between researchers and societal partners in the production of actionable knowledge, aimed at contributing to more equitable urban health.

Generating information and knowledge that is salient, timely, and understandable to decision-makers requires transdisciplinary dialogue between researchers from different academic disciplines and with decision-makers—including people who make choices in a variety of social contexts, from the household to government [2]. These dialogues start from an assumption that professional researchers are not the only producers of useful and legitimate knowledge. Many societal actors produce knowledge that is valid and useful [3]. Knowledge claims generated by scientific research is one form of knowledge that is widely distributed among social actors. The co-production of knowledge recognizes this distributed nature of knowledge and its production and organizes interactions between researchers and societal actors to encourage shared understanding of what is known and what can be done. We propose a way of framing the co-production of knowledge.

This paper has four sections. First, we set out core themes and concepts from the broad literature on co-production of knowledge. Second, we describe a ‘loops and building blocks’ framework for the practice of engagement between research, policy, and practice to generate valid and salient knowledge for action. Third, drawing on research in Accra, Ghana, we describe and assess co-production activities carried out in the *Pathways for Equitable Healthy Cities* project. Finally, we consider how co-production may be evaluated.

## Why co-Production? Key Themes and Core Concepts

The term ‘co-production of knowledge’ has emerged in a number of fields over the last 30 years, including public administration, science and technology studies (STS) and sustainability science [1], each developing a particular set of concerns and agendas. Elinor Ostrom, in her work on the provision of urban services like policing, defined co-production as a process by which inputs are transformed by individuals not in the same organization into goods and services [2]. The term was adopted by constructivist STS scholars in the 1980s as they sought to analyse the historical interplay between science and society [3, 4]. Since then, co-production has been used in a variety of ways, but the underlying concern with knowledge production as an active social process set in institutional and political contexts has remained.

Approaches to co-production range from normative to descriptive [5, 6]. We aim to contribute to the descriptive approach [7], which understands co-production as an interactive and complex process in which disciplines, practices, and knowledge systems can confront, shape, and be shaped by each other, whether by conflict or by cooperation. Diverse perspectives and understandings are reconfigured to generate new, transdisciplinary knowledge. In this process of interaction, new knowledge is intertwined with social, cultural, and political practices [5].

Given its mixed intellectual heritage, there is no unifying definition of knowledge co-production in the literature. We use the term co-production to mean a set of specific processes and practices that structure and organize the complex, interactive relationships between science, society, and policy. The benefits of co-production are clear and well-described, and include better quality of research, ownership and buy-in, accountability, empowerment, inclusion, and usability of knowledge [8–12].

Co-production has been explored in the field of public health, intersecting with a literature about ‘knowledge translation’ [13]. It has evolved alongside concepts of user participation and user input, and is often described as a way of working with patients, citizens, and organizations to design services that are more people-centred and user-led [8]. The value of co-production in health care has been widely explored [14] and there is broad acceptance of the value of processes involving multiple actors including patients, clinicians, managers, and carers aimed at jointly-creating knowledge, evaluating practice, or designing strategies [15–17].

Our framework is concerned with *learning*, which has emerged as a leading theme in STS co-production literature. Learning is seen as a long-term, evolving process, rather than a single event at a discrete moment. Co-production practices aim to create an environment in which learning occurs through dialogue, individual and joint reflection, and separate and joint experimentation with solutions [18, 19]. The process of active, structured dialogue points towards knowledge that is socially robust [20, 21]. This entails a shift to a conception of knowledge production as an open process, with multiple actors agreeing new knowledge claims and how they can affect action [22].

Co-production represents a multiple collaboration: between the sciences, between the different decision-makers, and between science and societal actors [23, 24]. This collaboration takes effort and, to participate, actors expect their effort to be rewarded. But the interests and incentives for will differ between participants. These differentiated incentives further complicate co-production, with the critique of ‘extractive’ research also being a concern for the conduct of engagement and dialogue [25].

In practice, not all knowledge claims are held to have equivalent value. A process of co-production often challenges existing hierarchies of value and esteem in knowledge systems. Knowledge is constructed against a background of social and institutional power relations and cultural factors [3]. Each knowledge claim draws on experience, assumptions, and expertise [26]. Since knowledge is socially embedded, co-production as a participatory process involving expert, practitioner, and lay knowledges may challenge conventional structures of knowledge production. This can entail tensions and conflicts. Co-production processes may therefore provide a space in which these tensions surface, are acknowledged, and, sometimes, resolved [27, 28]. Dealing with tensions is also a means of building trust. The production of knowledge, its appropriation, use, and misuse are processes set in gradients of power between organizations and people. New forms of information and knowledge governance acknowledging power relations are needed that mediate such challenges to existing systems of knowledge production [29].

## A Framework for co-Production: Loops and Building Blocks

The Pathways to Equitable Healthy Cities project (*Pathways*) is a global partnership that aims to improve population health, health equity, and environmental sustainability in cities through knowledge co-production with policy and civil society partners in cities in five countries (Accra, Tamale, Beijing, Dhaka, London, Vancouver). The project aims to produce evidence on how urban change and development can be shaped and managed to bring positive impacts to population health and health equity. The project works in cities of widely different economic, social, and health profiles. The project team was therefore challenged to develop a conceptual model and practical approach to knowledge co-production across contexts, building on previous examples.

**Fig. 1:**
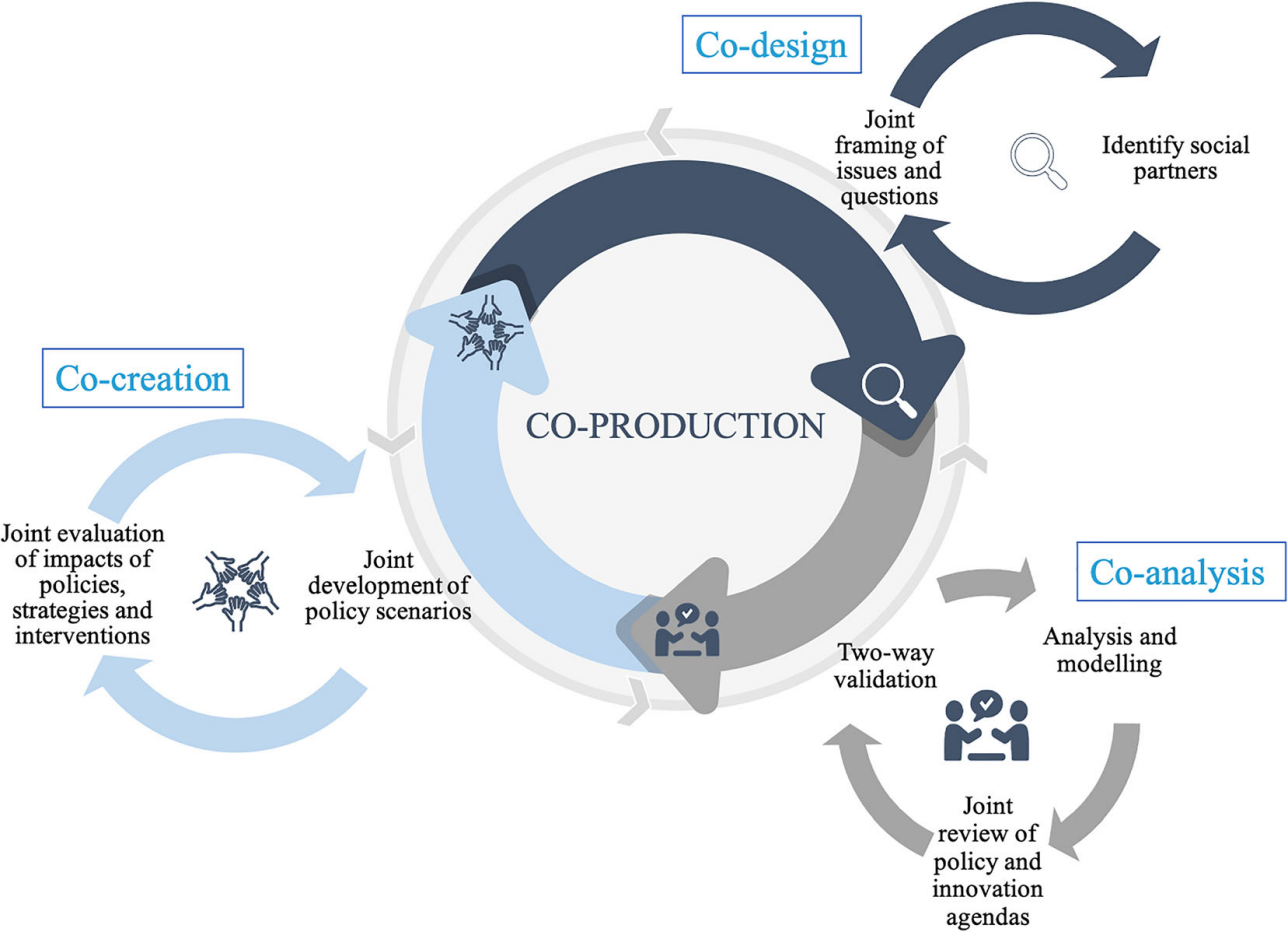
Conceptual framework for knowledge co-production developed for pathways.

The ‘loops and building blocks’ framework aims to structure more inclusive forms of engagement, knowledge production, and governance [28, 30] taking account of practitioner-oriented literature and experience in transdisciplinary projects [27, 31–33]. The framework defines co-production as including recursive three loops: *co-design*, *co-analysis*, and *co-creation* (see Fig. 1). Each loop represents a different stage of knowledge production. A first stage involves identifying partners and mapping their networks, and joint framing of problems and choices about the focus of research and action and agreement about common research and action objectives. This *co-design* loop allows early involvement of relevant societal partners and aims at trust-building, engagement and buy-in, open collaboration, and ultimately to project outputs becoming understood and influential to starting assumptions, choices, and decisions. The second stage (*co-analysis* loop) involves an agreed analytical strategy, the separate or joint collection of evidence, and analytical procedures, including modelling. Researchers and societal partners may work separately. Validation is at the core of this loop: through an exchange of information, researchers and partners validate each other’s work to achieve agreed knowledge claims in formats that are fitted to policy and practice design and implementation. Co-design and co-analysis are connected as new information or priorities may arise, leading to the identification of new partners and additional validation steps. The team will also jointly develop options and policy scenarios which identify interventions and actions to be tried out and evaluated [34]. The final *co-creation* loop includes implementation of policies, strategies, and interventions, typically in an experimental form, and their evaluation. This process may also lead to new knowledge and new, co-produced ideas for further research, leading to a new co-design loop.

In a situation of unconstrained time, resources, and commitment, it would be possible to move through each loop and between loops sequentially, recursively, and carefully. But research and policy projects are typically constrained in time and resources. Moreover, knowledge production in real-life action-oriented situations is messy, fluid, and contingent. Things change, partly in response to emerging findings and societal engagement. A flexible framework, able to adapt to unfolding events and opportunities, is therefore appropriate. Each loop is important, but the capacity to work through each one recursively may be constrained. Viewing co-design, co-analysis, and co-creation as a fixed sequence therefore seems too rigid. Instead, we see each loop as creating a new entry point for co-produced knowledge, and the time and effort placed in any given loop will vary between projects and action contexts. In this sense, knowledge co-production will always be *partial*: contingent on specific capacities and contexts, and therefore differing each time.

Given this freedom to apply comparable but varied approaches across the different contexts, projects need to recognize their own limits in time and resources to develop approaches that are both ambitious and realistic. In making choices about the balance of effort, projects need to develop a strategy for achieving impact. One of the greatest challenges for research is to provide knowledge claims in a timely way, aligned to the moments of opportunity when new evidence can have an impact in policy and practice [35]. Such moments of opportunity are often outside the gift of researchers to influence and they rarely occur when research has a completed, peer-reviewed result. Moreover, the problem that needs a solution may not match the problem which the research has sought to address. In the translation of co-produced knowledge to its use and adoption by decision-makers and practitioners, there is usually a degree of judgement and improvisation.

There is a second sense in which co-production of knowledge in a research project will be partial. Every project is embedded in an existing social context. Organized framing, analysis, and option generation that happens within a project (loops and building blocks) is always against a background of on-going framing, analysis, and experimentation elsewhere. Decision-makers and practitioners draw on a stream of competing knowledge claims in making choices and judgements [29].

A recognition of the partial and embedded nature of engagement organized within a research project does not imply a constraint on impact. The loops focus on distinct objectives in a broader process. Achieving these objectives increases the likelihood of impact as it creates buy-in by involving diverse actors, aligns outcomes and outputs to concrete needs, translates them into understandable languages, and ensures that they can be useful, usable, and by policy professionals and practitioners.

Many research projects last three to five years and may be carried out by cross-country consortia entailing different cultures, contexts, and languages and encompassing different partners (NGOs, government bodies, private sector, civil society representatives, etc.). Embedding a co-production framework in such contexts has proved to be challenging and may lead to unrealistic goals. However, project reviews suggest that even single workshops, informal interactions, and feedback in either direction can produce learning by researchers and societal partners [32]. For this reason, we argue for a realistic approach, matching the resources and incentives of all participants in the research. We believe that a process of organized co-production needs to be adaptive and flexible, picking up dynamically on opportunities as they arise during the flow of a project. The aim should be to maximize inclusion of diverse and weak voices, and to challenge the dominance of specific perspectives, increasing epistemic diversity. This is in line with most recent literature which places weight on systemic changes in the governance of knowledge production [28–30].

‘Building blocks’ are the practical expression of co-production loops. We define a building block as any time-bound activity, principle, interaction-based method, or tool aimed at bringing together a range of participants involved in research to work together on a specific task: framing, analysing, or designing and evaluating interventions. Carter et al. introduce the idea of building blocks in the context of co-production of climate and weather services in Africa [27]. We have developed this idea and embedded it in our framework. A building block could be a workshop, a webinar, or collaboration with a government agency to secure health or population data. We propose a modular construction of building blocks, activated in the broader context of the three co-production loops, which themselves are conceived of as operating against a wider societal background of knowledge production and action. Different building blocks would be appropriate to different loops, as shown in Table 1 below. Making loops and building blocks explicit helps address the question of comparability across urban contexts and equitable, health in cities, highlighting the ‘one-size does *not* fit all’ approach, while providing a framework for comparative analysis and evaluation across divergent contexts.

**Table 1:**
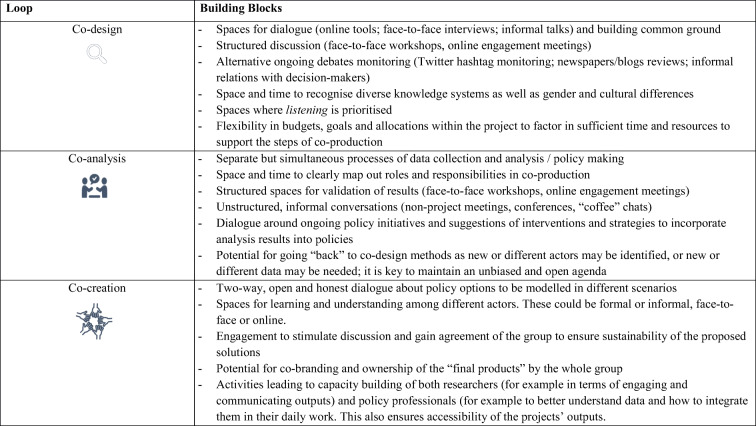
Building block examples for each of the loops in pathways. Adapted from Carter et al. (2019).

## Loops and Building Blocks in Practice

Co-production in research has benefits when used to influence decision-makers. It is a time and resource-intensive process that requires commitment from all involved parties. Because of this, the incentives for participation by researchers and partners need to be clarified: what is in it for each side? [33]. The objective of *Pathways* is to provide timely, rigorous, and scientific evidence on urban change and development to evaluate how urban and health policies will impact the urban environment and population health, through a lens of health equity. Influencing urban planning to improve health equity requires a focus on policies. This translates into interactions between researchers from different disciplines with policy professionals, government officials, and civil society organizations. Such an approach favours policy professionals and ‘elite’ stakeholders over direct engagement with citizens and other decision-makers, to prioritize inputs and influence over national- and local-level policies affecting urban health.

## Who Is co-Producing Knowledge? A Building Block from Accra, Ghana

In the initial co-design phase of the project, we convened two face-to-face workshops in Ghana in 2019. This followed more informal consultations with project partners at the University of Ghana during project development. The two workshops were convened in Accra, Ghana’s capital [36]. The first workshop, held in May 2019, was a context-setting session where partners from the Accra Metropolitan Assembly, the main political and administrative authority for the city, the large civil society organization *People’s Dialogue on Human Settlements* (https://www.pdghana.org/), and representatives from Ghana Statistical Service spoke about urban planning and health issues and priorities to researchers. Sixteen policy professionals and opinion leaders were invited, and an open dialogue was organized in small groups involving fourteen researchers collaborating in *Pathways*. Focussing on attentive listening by researchers, the session aimed to reverse the conventional relationship between researchers, and policy makers and activists. Here the societal partners were given time to elaborate on their own perspectives on the relationship between urban development and health in Accra without any framing by researchers.

The informal setting allowed policy makers and activists to tell rich stories about the rapid and extensive growth of the city, the goals of the structure plan for the Greater Accra Metropolitan Area, day-to-day problems of governance and administrative capacity, and the important role played by citizen’s organizations and the private sector in shaping urban change. This provided perspectives for researchers of policy priorities, place attachment, ethnic challenges, social dimensions, economic trends, and equity, and how these are discussed in the context of contemporary Accra. This example is useful in showing that, while the act of listening may seem like a one-way process, it can create a space for reflection and learning that is the basis for co-production. It embodies perspectives, knowledge, and positionality in real people and places.

For the second more intensive workshop, in October 2019, we used the Participatory Impact Pathways Analysis (PIPA) approach to discuss issues, priorities, and ways forward in urban development and planning. PIPA is a project planning and research methodology, developed at the International Centre for Tropical Agriculture from about 2005. It can be adapted to different contexts and aims, and is designed to help the people involved in a project, program, or organization to make explicit how they see themselves achieving their goals and impact [37]. The aim of this workshop was to build common ground and create a shared vision of challenges and opportunities for urban planning and health equity in Accra. PIPA engages societal partners in a structured participatory manner, allowing time for reflection and promoting learning.

In Accra, the team focused on how the project would develop its research outputs and who outside the project would need to use them to achieve developmental outcomes. In practice, this meant discussing specific fields of urban development (transport, water, sanitation, air pollution, noise pollution, density, land tenure…), what they meant for the policy professionals in the room, how they were prioritized, and what the *Pathways* project could do to support policymaking in these areas. Over the course of the two days, participants were asked to design ‘problem trees’ to specify issues that *Pathways* researchers could, realistically, help to tackle in Accra. Drawing from the more specific, easier to tackle issues, participants were then asked to envisage a short- to medium-term vision of the city. In this visioning exercise, they were asked to keep the discussions realistic, guided by a timeline of changes that could be achieved in the project’s time frame.

This was followed by a network mapping session seeking reflection on which actors, authorities, decision-makers, policy professionals, and citizens should be involved in making change happen, as well as existing or missing links between them. This discussion created awareness among researchers of the complex networks of relevant actors and their multiple relationships. The network of actors considerably extended the scope and complexity of change to be envisaged from *Pathways* research, while also increasing the range of potential partners for the project. Finally, the participants were asked to articulate action plans to reflect on the development trajectory of Accra and what identified actors would need to do. Deep policy already exists in many of the domains that were mentioned (e.g., upgrading services in slums, land tenure, public transport, waste management, and vehicle emissions), with participants agreeing that the real challenge is often to implement what is already on paper. This point about governance capacity confirmed the need for the project to be deeply engaged with the existing policy context, including the potential for critical linking interventions to unlock implementation of plans.

As a result of this workshop, *Pathways* priorities and workplans shifted, in particular by reallocating attention more towards the topic of housing and land tenure. Researchers agreed to look into urban planning trade-offs and the provision of health-related services in different scenarios, as well as potential impacts on health and equity. Researchers were later debriefed in person, via email, and via an online questionnaire and asked to reflect on how this workshop had affected the way they were carrying out their work. These mini evaluations showed that the workshop encouraged participants to reflect beyond the scope of the project and work to establish interpersonal and organizational links that would be needed for longer-term impact. Researchers also highlighted that it gave them an opportunity to create or strengthen networks and relationships with specific actors, government representatives, or NGO workers who have since been closely involved with working groups in the project. This exemplifies the modular conceptualisation of co-production and shows how different actors can add building blocks, giving the process more depth and breadth.

### Engaging with the Wider Context: Monitoring Twitter

The *Pathways* project made a conscious choice early on to target policy professionals. However, co-production aims to be inclusive. In an urban context of equitable health, this should also include citizens and people living or working in the city and its surroundings. To address the gap in community-level engagement and to assess ongoing public and policy discourse, the project began to monitor social media commentary on urban development and health. Starting in Accra with housing and health issues, we initiated a systematic and quasi-automated web-scraping system for monitoring Twitter commentary using simple lines of code in Python to collect data from Twitter’s application programming interface (API). Tweets are gathered and coded in qualitative analysis software to allow text, discourse, or narrative analysis.

We assume that Twitter commentary partially reflects and could potentially shape how urban and health problems are framed. We aim to investigate patterns of these debates in Accra, including their evolution through time, summarizing them as an input for research across the project and a background to communication and impact-oriented work. This building block offers the opportunity to identify other partners and more priorities that could feed in the researchers and policy professionals’ agenda and add a social dimension to our agenda, especially at a time when direct interactions with local people are made harder by the global pandemic.

## Monitoring and Evaluating co-Production

Monitoring and evaluation of engagement in knowledge co-production presents many challenges. There are multiple interactions between project partners with a wide variety of purposes and encompassing diverse disciplines and societal actors; linking each of these to eventual project outcomes will be difficult, also given the existence of multiple alternative factors explaining these outcomes. Moreover, the problem of evaluation of small-n, local, and experimental interventions is well-understood [38]. There is likely to be a balance between conventional scientific notions of rigour in evaluation (randomized control trials, for instance) and more hybrid, evaluative approaches, including policymakers and practitioners.

A monitoring and evaluation framework would ideally be continuous and in real time, allowing project partners to learn and adjust as a project proceeds. Each loop may invite an evaluation before moving on. In such a process, learning is integrated in the project and evaluation becomes intrinsic to co-production loops and building blocks. Previous projects have identified multi-directional learning as raising the likelihood of success and sustainability of co-production processes and outcomes [27, 39]. For co-production to be evaluable and effective, projects need built-in feedbacks to allow for course adjustments [39]. The context of the research should itself guide success criteria, reflecting the distributed nature of co-production, as well as the diverse interests of the actors. The co-production process is often overlooked by funders in performance metrics. Similarly, it is often the case that researchers lack incentives to carry out co-production and prioritize academic outputs to engagement. However, some processes such as behavioural changes in scientists, trust among actors across different disciplines, or changes in hierarchies and relationships, can be assessed before the co-produced product is developed by setting up mechanisms such as key informant interviews over the course of the project. In *Pathways*, brief online Google Forms are set up for researchers across the project to keep track of their interactions. This keeps the teams accountable for their outreach, but also allows our research to focus on comparability across cities.

## Conclusions

This paper puts forward a practical and flexible ‘loops and building blocks’ approach for co-production processes which draws on previous research in a diverse range of research fields and aims to support engagement between researchers and societal partners. Co-production encompasses the complex process of formal and informal interactions between researchers of different backgrounds, disciplines, and institutions, and between researchers and policy professionals, decision-makers, and other social actors. This paper offers some practical ways of creating this awareness, encouraging a flexible and adaptive approach including ongoing monitoring and adjustment, supporting through potential obstacles, and creating outputs that are relevant, usable, and useful by practitioners and decision-makers.

Co-production is a process. Having established that there can be no ‘one size fits all’ approach, the framework we offer can be adjusted to the objectives, resources, and interests of research, and fitted to the needs of specific contexts and social dynamics. By developing a monitoring, evaluation, and learning framework, we seek to encourage the application of successful co-production tools and approaches in new contexts at different scales. This largely depends on participants finding value in the process and integrating it further into their work but ultimately leads to sustainability.

It is important to keep in mind that a co-production process is always intervening over a background of ongoing relationships and discourses that existed well before the project and will persist beyond its completion. Moreover, it is also key to remember that co-production demands commitment, time, and resources by people who engaged in it. To ensure continued buy-in and engagement, all participants, including researchers, need incentives and rewards for these investments. If research is usually a collaborative effort, then achieving change through new knowledge production in research involves an extended form of collaboration, with shared benefits. This is effortful and will only be effective if it achieves the right balance between being planned, flexible, and opportunistic.

The COVID-19 pandemic presents both challenges and opportunities to co-production processes and thinking. The absence of face-to-face interactions may significantly hinder co-creation processes aiming at creating common ground and building trust among different parties. On the other hand, greater familiarity with virtual meetings and with more pervasive use of social media, there are also new and more efficient modes of digital engagement and data collection. An adaptable and modular loops-and-blocks framework can be flexible to these new realities. There is an opportunity to think beyond face-to-face engagement and explore new opportunities for remote outreach.
